# Chemical glycobiology

**DOI:** 10.3762/bjoc.21.2

**Published:** 2025-01-03

**Authors:** Elisa Fadda, Rachel Hevey, Benjamin Schumann, Ulrika Westerlind

**Affiliations:** 1 School of Biological Sciences, University of Southampton, Southampton SO17 1BJ, United Kingdom; 2 Department of Pharmaceutical Sciences, University of Basel, 4056 Basel, Switzerland; 3 Department of Chemistry, Imperial College London, London W12 0BZ, United Kingdom; 4 Chemical Glycobiology Laboratory, The Francis Crick Institute, London NW1 1AT, United Kingdom; 5 Department of Chemistry, Umeå University, 90736 Umeå, Sweden

As glycoscientists, we are standing on the shoulders of giants. Research on carbohydrates is as old as on any other biomolecule, dating back to the time of Emil Fischer and the elucidation of monosaccharide structures [1]. Later, foundational contributions came in the form of the first glycoconjugate vaccines [2–3], the elucidation of the blood group system [4], and many others. Among these, we dare to include the DNA double helix, featuring deoxyribose as a key structural element of its twisting ladder [5]. A century of innovation, some of the most prestigious awards and highest honours later, one aspect is immediately clear: chemistry and glycobiology are intricately intertwined. This is certainly by choice, but also by necessity. It is difficult to convey to non-glycoscientists how we still struggle with challenges that have been solved years or decades ago for proteins and nucleic acids. When molecular cloning and recombinant protein production became routine, these technologies were not applicable to glycans. Today, the most amazing tools in genome engineering are used to great effect to disrupt or alter the glycan biosynthetic machinery, but they still cannot be used to, for instance, mutate one glycan into another in the same manner as nucleic acids can be mutated. Methods in molecular biology are facile and quantitative. But they do not tell us the function of a particular glycoform on a specific glycoprotein. To put glycans on the map, chemists needed to be inventive.

At the time of writing this Editorial article, we are all early- and mid-career investigators who have learned from the best. We look in awe at the achievements in the field to date, some of those appearing in the previous thematic issues “GlycoBioinformatics” [6] and “Synthesis in the glycosciences” I and II [7–8]. We look ahead, asking the question how we can implement new chemistry, new molecules, and new methods to make the glycosciences even more palatable to generalists. And we see a field that innovates.

This thematic issue seeks to highlight the amazing breadth of contemporary chemical glycobiology. Dal Colle et al. investigate the determinants that influence the oligosaccharide yield in automated glycan assembly [9]. Target-directed synthetic strategies are being developed by Reihill et al. [10] and Karak et al. [11], exploring the syntheses of the linker-displaying, sulfated TF disaccharide and lipid II analogues, respectively. The direct application of synthetic glycans is shown by Fan et al. [12] in the context of photoswitchable ligands to the lectin LecA. Staying in the theme of lectin characterization, Lundstrøm et al. study the glycan binding profile of CMA1 originating from melon [13].

A time that sees great opportunities in computational biology also breeds innovative applications in the glycosciences. A key aspect is the modelling of protein–glycan interactions. Marcisz et al. study the power of umbrella sampling in distinguishing the interactions between different glycosaminoglycans and their receptors [14]. Nieto-Fabregat et al. provide a detailed overview on computational methods that underlie modern glycobioinformatics approaches [15]. Validation of glycoprotein structure is an important aspect of contemporary structural biology, and Dialpuri et al. present the Privateer database to allow for facile quality control of such structures [16]. Finally, Barillot et al. bridge experimental and computational efforts, developing a neural-network-based approach for the interpretation of glycan structures from their vibrational fingerprints [17].

We anticipate that this diverse collection of reports across the entire spectrum of the chemical sciences cements the readers’ understanding of chemistry as being a catalyst to more than a century of glycobiology, with a profound and exciting vision for the future.

Elisa Fadda, Rachel Hevey, Benjamin Schumann and Ulrika Westerlind

Southampton, Basel, London, Umeå, November 2024

## Data Availability

Data sharing is not applicable as no new data was generated or analyzed in this study.
